# Nanocellulose Reinforced Hyaluronan-Based Bioinks

**DOI:** 10.1021/acs.biomac.3c00168

**Published:** 2023-06-21

**Authors:** Andrea Träger, Sajjad Naeimipour, Michael Jury, Robert Selegård, Daniel Aili

**Affiliations:** Laboratory of Molecular Materials, Division of Biophysics and Bioengineering, Department of Physics, Chemistry and Biology, Linköping University, 581 83 Linköping, Sweden

## Abstract

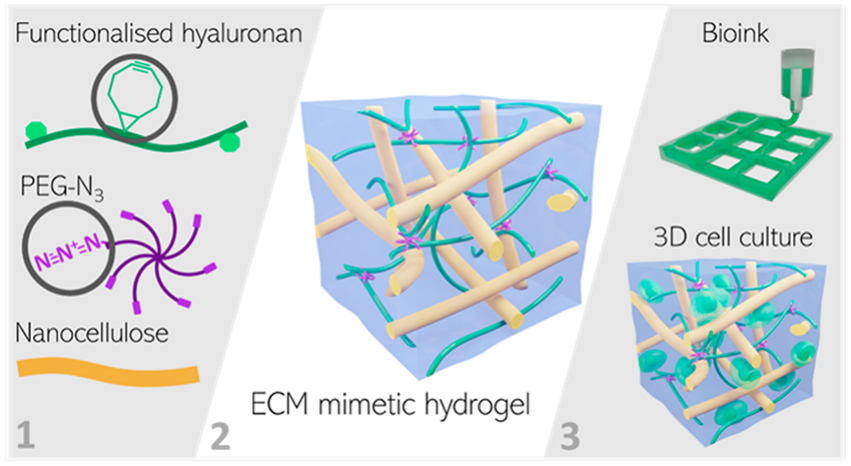

Bioprinting of hydrogel-based bioinks can allow for the fabrication of elaborate, cell-laden 3D structures. In addition to providing an adequate extracellular matrix mimetic environment and high cell viability, the hydrogels must offer facile extrusion through the printing nozzle and retain the shape of the printed structure. We demonstrate a strategy to incorporate cellulose oxalate nanofibrils in hyaluronan-based hydrogels to generate shear thinning bioinks that allowed for printing of free-standing multilayer structures, covalently cross-linked after bioprinting, yielding long-term stability. The storage modulus of the hydrogels was tunable between 0.5 and 1.5 kPa. The nanocellulose containing hydrogels showed good biocompatibility, with viability of primary human dermal fibroblasts above 80% at day 7 after seeding. The cells were also shown to tolerate the printing process well, with viability above 80% 24 h after printing. We anticipate that this hydrogel system can find broad use as a bioink to produce complex geometries that can support cell growth.

## Introduction

Hydrogel-based three-dimensional (3D) cell culture has garnered steadily increasing interest as it affords the possibility to grow cells in a geometry more similar to their native microenvironment compared to conventional two-dimensional (2D) systems. 2D cell culture substrates are typically rigid coated plastic surfaces, which lack the ques driving many aspects of cellular behavior, such as proliferation,^1^ differentiation,^2^ and migration.^3,4^ An optimized 3D cell culture scaffold can mitigate many of these drawbacks. In addition to a favorable geometry, hydrogels used for a 3D cell culture can provide the structural and chemical components that are integral for cells to thrive.^1^ Hydrogels based on harvested extracellular matrices (ECMs)^5,6^ feature the full range of ECM proteins and fibrils. However, due to the complex composition and biological origin of these materials, they are very challenging to characterize and tailor to specific needs. Furthermore, they tend to suffer from large batch-to-batch variation,^7^ leading to poor experimental reproducibility. Not to mention that they are expensive to produce.^1,7^ Engineered ECM-mimetic hydrogels are interesting alternatives to biosourced ECM, since they allow full control of the included components and high reproducibility with respect to both chemical and mechanical properties. Engineered ECM inspired hydrogels will always have a less complex composition than native ECM, but their properties can be tailored to promote biologically relevant cellular behavior.^8^

The advances in 3D cell culture have promoted the use of hydrogel-based scaffolds in several emerging applications, such as drug screening,^9,10^ regenerative medicine,^11,12^ and 3D bioprinting.^13^ For bioprinting, the hydrogels not only must provide a biologically relevant microenvironment but also exhibit rheological properties that support the printing process. Polymer solutions with low viscosity may not maintain an even distribution of cells within the bioink cartridge during the printing process. K. Na et al. found the cell sedimentation to reduce significantly when comparing bioinks with viscosities of 0.003 and 60 Pa·s.^14^ Another challenge with low viscosity bioinks is that they may not retain the printed structure, resulting in a poor printing resolution. One approach to address this issue is to increase the viscosity of the bioinks to mitigate cell sedimentation and improve the resolution. However, the high viscosity requires higher extrusion pressure, which can potentially be harmful to cells.^15^ Shear thinning hydrogels are attractive components in extrusion bioinks, since this facilitates the dispensing procedure while allowing for printing with high shape fidelity.^16^ As the name implies, the viscosity of a shear thinning material will decrease under shear stress, which can reduce the impact of shear forces generated during the extrusion process on the cells, while the higher viscosity at rest benefits shape fidelity and resolution of the printed structures after extrusion. A range of macromolecules, of both biological and synthetic origin, have been explored as structural components in ECM mimetic hydrogels for bioprinting, such as collagen,^17^ alginate,^18^ hyaluronic acid (HA),^19^ polyethylene glycol (PEG),^20^ silk fibroin,^18^ and cellulose.^21^ Cellulose is an abundant biopolymer with excellent cytocompatibility^22^ that can be tailored for a wide range of applications.^23−26^ Nanocellulose, where the cellulose fibers have been separated into structures with at least one dimension in the nanometer range, are explored for numerous applications, ranging from energy storage devices^25^ to reinforcement in composites^23^ to biomedicine.^24^

In addition to rheological properties that support cell viability throughout the printing process and shape fidelity immediately after printing, the printed structure must maintain high structural stability throughout the time frame in which the printed structure is intended to be used. This is commonly achieved by some mode of cross-linking. The cross-linking procedure should ideally impede neither the extrusion process nor cell viability. Inclusion of a cross-linker in the printing cartridge restricts both the printing time and size since the fully cross-linked material will not be able to flow. A common method of postdeposition cross-linking is to use photoinitiators in combination with UV light, which can have cytotoxic effects.^27^ In contrast, noncovalent strategies to form linkages in the gel, such as relying on hydrophobic interactions or hydrogen bonding, is often well tolerated by cells but tends to result in hydrogels with relatively low stability over time and under varying conditions.^27^ An attractive option is covalent cross-linking post extrusion through a bioorthogonal reaction.

**Figure 1:**
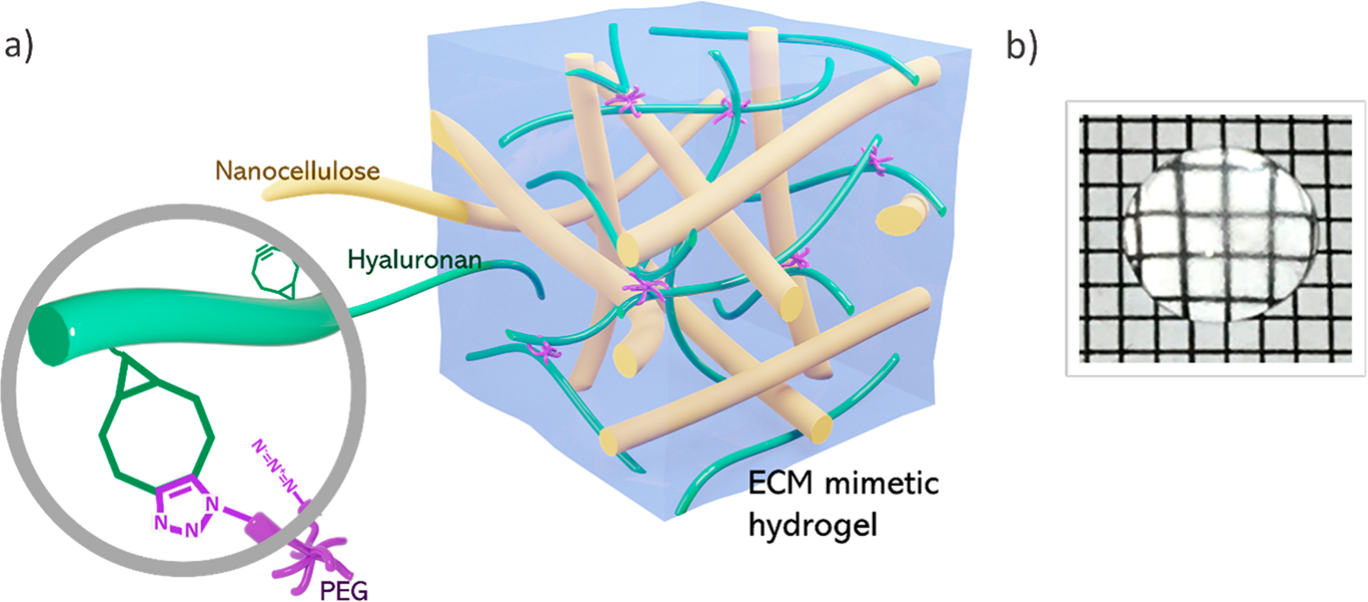
(a) Schematic illustration of the nanocellulose reinforced hyaluronan-based hydrogels (HA-PEG). The BCN-functionalized hyaluronan is cross-linked by eight-arm PEG-azide (PEG-Az_8_) through SPAAC, trapping the nanocellulose in the polymer network. Not to scale. (b) Photograph of a nanocellulose reinforced HA-PEG hydrogel placed on a grid pattern where each square represents 1 × 1 mm.

We have recently developed ECM mimetic hydrogels based on cyclooctyne-functionalized HA (HA-BCN) cross-linked through a strain-promoted azide–alkyne cycloaddition (SPAAC) reaction using multiarm azide-functionalized PEG (PEG-Az_8_).^28,29^ In addition to allow for bioorthogonal cross-linking of the hydrogels, unreacted BCN groups can be utilized for functionalization of the HA with cell adhesion motifs or other functional groups.^8^ This versatile hydrogel system has been demonstrated to support the proliferation of several cell lines such as fibroblasts,^28^ hepatocytes,^29^ and neural cells^28^ and has been utilized as a bioink for 3D bioprinting.^28^ However, since the cross-linking commences immediately after the cross-linker (PEG-Az_8_) has been added to HA-BCN, the printing window, i.e. when the hydrogel can be extruded, is limited to a few minutes. Printing too early, before the hydrogel is sufficiently viscous, results in low printing resolution. Waiting too long, on the other hand, results in too extensive cross-linking and obstruction of the extrusion nozzle. This is an issue seen with many hydrogel systems where cross-linking is initiated upon mixing of the components,^30^ and which can significantly complicate bioprinting. In this paper, we present a strategy to enhance the printability of this hydrogel system by the incorporation of cellulose nanofibrils (Figure 1), generating high zero shear viscosity hydrogels with a large printing window that both support cell proliferation and allow for convenient bioprinting.

## Experimental Section

### Nanocellulose dispersions

: Nanocellulose dispersion prepared as previously described^31^ was kindly provided by the company FineCell Sweden AB (Linköping, Sweden). For the sample named NC_0_, the cellulose oxalate powder was dialyzed against Milli-Q water before homogenization. For the samples NC_15_ and NC_60_, the cellulose dispersion was diluted 10× by Milli-Q, placed in a round-bottom flask equipped with magnetic stirrer, which was placed in an oil bath preheated to 115 °C, and left there with stirring for 15 and 60 min, respectively. Then, NC_15_ and NC_60_ were again dialyzed against Milli-Q water and centrifuged at 4500 rpm for 60 min at 10 °C, after which the supernatant was discarded. The dry content of the nanocellulose dispersions was determined by placing a preweighed sample in an oven at 90 °C overnight and then comparing the dry weight to the initial sample weight. Charge density of the nanocellulose dispersion was determined through conductometric titration, as previously described.^32^*Synthesis of HA-BCN*: The synthesis of HA-BCN was performed as previously described.^33^ In short, HA (100–150 kDa, Lifecore Biomedical) was dissolved in MES buffer (100 mM, pH 7) and *N*-[(1R,8S,9S)-bicyclo[6.1.0]non-4-yn-9-ylmethyloxycarbonyl]-1,8-diamino-3,6-dioxaoctane (BCN-NH_2_) dissolved in a 5:1 (v/v) acetonitrile/water mixture prior to addition of 1-ethyl-3-[3-(dimethylamino)propyl]-carbodiimide and 1-hydroxybenzotriazolehydrate. This solution was then added to the HA. The carbodiimide cross-linking reaction was allowed to proceed for 24 h, followed by dialysis in acetonitrile/water (1:10 v/v) for 24 h, followed by Milli-Q water for 3 days before lyophilization. *Hydrogel formation*: HA-BCN, PEG-Az_8_ (8-armed poly(ethylene glycol) aizde, 10 kg/mol, from Creative PEGworks, Chapel Hill, NC, U.S.A.), and nanocellulose dispersions were UV sterilized (60 kJ/cm^2^) for 1 min. HA-BCN and PEG-Az_8_ were dissolved in PBS or a cell culture medium. The cellulose dispersion was diluted as required by the requisite amount of Milli-Q water to achieve the desired concentration. The HA-BCN solution was mixed with nanocellulose dispersion (or Milli-Q water when preparing a gel without nanocellulose). This HA-BCN:nanocellulose solution was then mixed with the PEG-Az_8_ solution, aiming for a BCN/N_3_ ratio of 10:1, with the exception of material use for bioprinting. In the latter case, the PEG-Az_8_ solution was added after printing a 3D structure of the HA-BCN/nanocellulose mixture. *Hydrogel characterization*: The mechanical properties of the hydrogels were evaluated through oscillatory rheology using a Discovery HR-2 rheometer, TA Instruments. Preformed hydrogels swollen in PBS buffer overnight were evaluated for a minimum of quadruplicate samples at room temperature using an 8 mm plate geometry with frequency sweeps at a fixed oscillatory strain of 1%, and amplitude sweeps at a fixed frequency of 1 Hz. Gelation kinetics was evaluated for at least duplicate samples with a 20 mm 1° cone–plate geometry at 0.1% strain and an oscillatory frequency of 1 Hz. The components of the hydrogel were mixed and immediately placed on the sample holder at 4 °C. After bringing the geometry into position and applying a solvent trap to avoid dehydration of the hydrogel, the temperature of the sample holder was increased to 37 °C and the measurement started. Creep-recovery tests were performed in a manner similar to the gelation kinetics experiments, but at room temperature. When the samples had cross-linked fully, a constant shear of 50 Pa was applied for 60 min, and strain changes recorded. Then, the shear was released, and strain was recorded for another 75 min. *SEM*: Samples were prepared for scanning electron microscopy (SEM) by immersion in N_2_(*l*) followed by lyophilization, mounted on a carbon grid, and finally sputtering with Pt. SEM measurements were performed on a LEO 1550 Gemini (Zeiss) operating at 5 kV. *Cell viability*: Primary human fibroblasts obtained from skin biopsies from healthy patients were kindly provided by Dr Johan Junker (Linköping University, Sweden). All experiments involving human tissue and cells were performed under ethical approval from the Swedish Ethical Review Authority (no. 2018/97–31) and in accordance with ethical standards postulated by Linköping University and Swedish and European regulations. Briefly, deidentified skin samples from heathly female patients undergoing routine surgery were repeatedly washed in sterile PBS and subcutaneous fat and epidermis were mechanically remove. The dermis was enzymatically dissociated with 165 U/mL collagenase (Gibco Thermo Fisher Scientifik, U.K.) and 2.5 mg/mL Dispase (Gibco Thermo Fisher Scientifi, U.K.) and incubated at 37 °C, 5% CO_2_, 95% humidity overnight. Samples were centrifuged for 5 min at 365×g. The cell pellet was resuspended in fibroblast medium (Dulbecco’s modified Eagle medium, DMEM, with 10% fetal calf serum, 50 U/mL penicillin, and 50 mg/mL streptomycin). The isolated fibroblasts were cultured in DMEM high glucose (Biowest) and supplemented with 10% fetal bovine serum (FBS) and 1% penicillin/streptomycin (P/S). Fibroblasts used in this study were in passages 5–8. SH-SH5Y cells (ATCC CRL-2266) were cultured in Biowest DMEM-F12, supplemented with 10% FBS, 1% P/S, and 1% nonessential amino acids. The cells were trypsinized, centrifuged, and resuspended in the PEG-Az_8_ solution. After mixing the PEG-Az_8_ solution with the other components of the hydrogel, the mixture was transferred to a sterile 96 well plate, 25 μL per well, after which the well plate was left in the incubator at 37 °C for 1 h before addition of the cell culture medium. Cell viability was evaluated either using alamarBlue (Thermo Fisher) or by staining with a Live/Dead Viability/Cytotoxicity Kit (Thermo Fisher), and cells were imaged using a confocal microscope (Zeiss LSM 700). The confocal images of live and dead cells were evaluated by using ImageJ, where the number of cells on each image was manually counted. A minimum of three images were counted and averaged for each condition. *3D bioprinting*: The bioink was prepared as described under “*Hydrogel formation*”. Bioprinting was performed using a BioX printer (Cellink AB, Gothenburg, Sweden) at 7 kPa pressure and a nozzle speed of 10 mm/s, with 50 ms postflow delay. To print a Linköping University logo, 2–3 mL of ink was loaded into a cartridge with a red dispensing tip (gauge 25). For cell experiments, the bioink was gently mixed with the cells and 100 μL of the HA-BCN/nanocellulose bioink was added to the back of a blue dispensing tip (gauge 22) to print a 10 × 10 × 3 mm lattice structure. After printing, the sample was cross-linked by gently applying 1% (w/v) PEG-Az_8_ dissolved in PBS or cell culture media. The samples were kept in the PEG-Az_8_ solution at 37 °C for 90 min before being transferred to the storage solution, either isotonic NaCl, PBS, or cell culture media.

## Results and Discussion

### Nanocellulose Dispersions

The nanocellulose dispersions used in this study consisted of cellulose oxalate. To study the influence of the charge density on the rheological properties and cell viability of the resulting hydrogels, samples of three different charge densities were prepared by removing oxalate groups from the initial nanocellulose sample (NC_0_) through hydrolysis. This yielded samples NC_15_ and NC_60_, where the subscripts represent the duration of the hydrolysis step. The charge densities of the three cellulose dispersions were evaluated through conductometric titration on duplicate samples for each dispersion and measured 107, 127, and 207 μequiv/g for dispersions NC_60_, NC_15_, and NC_0_, respectively. These numbers corroborate a difference in degree of functionalization regarding oxalate side groups and that the hydrolysis of NC_15_ and NC_60_ was successful. The measured charge density for NC_0_ is in agreement with previous measurements by FineCell on their cellulose oxalate dispersions (unpublished data).

### Nanocellulose Reinforced Hydrogels

**Figure 2:**
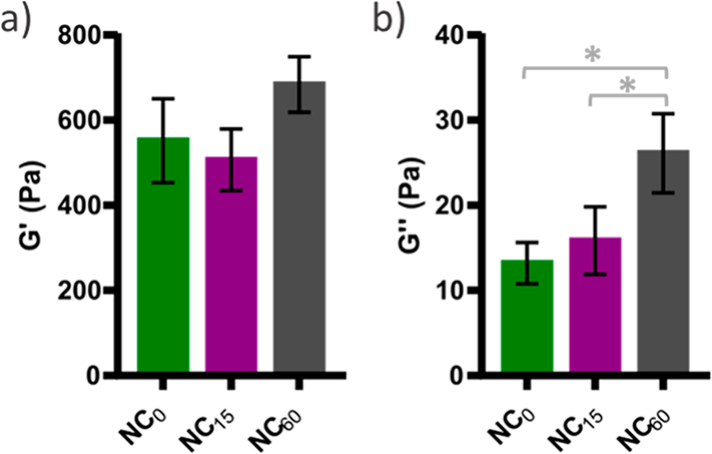
Storage (*G*′) and loss (*G*′′) moduli at a frequency of 1 Hz and oscillation strain of 1% for 1 w/w% HA-PEG hydrogels containing 0.5 w/w% of either NC_0_, NC_15_, or NC_60_. Reported values are averages of at least four replicates, with error bars showing standard deviations. *p < 0.05 (ANOVA with posthoc Tukey HSD).

HA-PEG hydrogels were obtained by cross-linking HA-BCN by PEG-Az_8_ through SPAAC as previously described.^28,29^ To evaluate the effect of nanocellulose charge density on the rheological properties of the hydrogels, 1% (w/v) HA-PEG was prepared, containing 0.5% (w/v) of NC_0_, NC_15_, or NC_60_ and evaluated by oscillatory rheology (Figure 2). The difference in rheological properties between these hydrogels was very small. There was a slight but significant (*p* < 0.05) increase in loss modulus (*G*′′) for the hydrogel containing NC_60_ compared to NC_0_ and NC_15_. Given the very small difference in the rheological properties, subsequent rheological measurements were performed only with NC_0_. The choice fell on NC_0_ since the nanocellulose was well dispersed during the homogenization procedure, and the less it was processed after homogenization, the lower was the tendency of the dispersed fibrils to aggregate.

**Figure 3:**
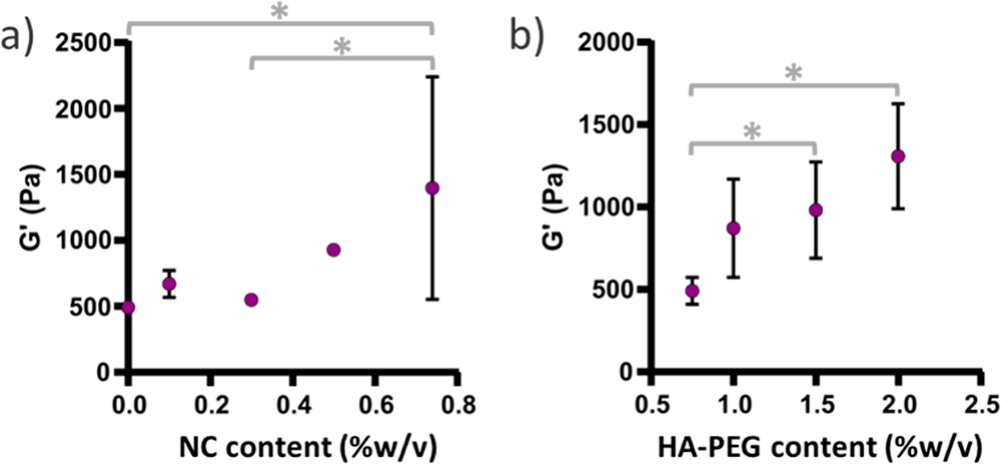
(a) Storage modulus at 1 Hz and 1% strain of 2% (w/v) HA-PEG hydrogels containing between 0 and 0.7% (w/v) NC_0_. (b) Storage modulus at 1 Hz and 1% strain of hydrogels with 0.5% (w/v) NC_0_ containing different concentrations of HA-PEG. Reported values are averages of at least four measurements. *p < 0.05 (ANOVA with posthoc Tukey HSD).

Comparing the rheological properties of 2% (w/v) HA-PEG hydrogels containing between 0 and 0.7% (w/v) of NC_0_ (Figure 3a), the addition of 0.1–0.3 w/w% NC_0_ had little to no effect on the storage modulus, whereas there was an increase in the storage modulus of the resulting hydrogel for NC_0_ concentration above 0.3% (w/v). The tan delta and loss modulus increased with approximately 1 and 2 orders of magnitude, respectively, when increasing the NC_0_ concentration from 0 to 0.7% (w/v) NC_0_ (Figure S1). The effect of different concentrations of HA-PEG on the rheological properties was also evaluated. Figure 3b shows an increase in the storage modulus with increasing HA-PEG content. The tan delta was somewhat higher for the sample with the lowest HA-PEG content compared to that for the other three compositions (Figure S2), which can be a result of the cellulose having a larger influence on the viscoelastic properties of this sample given the low HA-PEG content. These observations indicate that the dispersed nanocellulose forms an entangled network within the covalent HA-based hydrogel that efficiently dissipates energy, resulting in shear thinning hydrogels.

**Figure 4:**
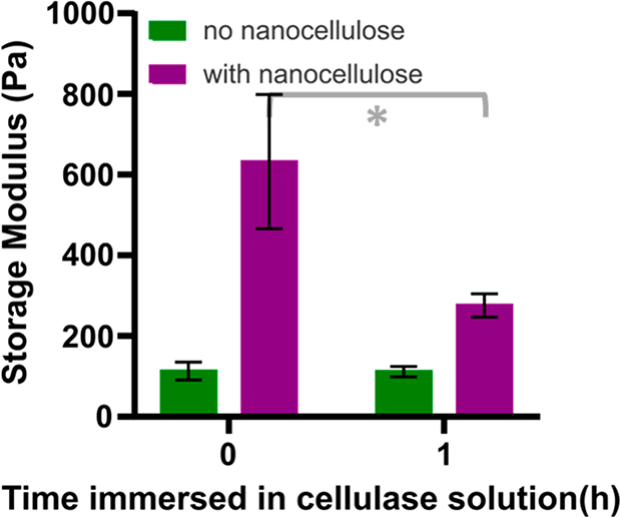
Storage modulus at 1 Hz frequency and 1% oscillation strain of 0.75% (w/v) HA-PEG hydrogels with and without 0.5% (w/v) NC_0_ immersed in a pH 5 acetate buffer at 37 °C containing 100 mM NaCl, before and 1 h after addition of 3.3 mg/mL cellulase to the acetate buffer solution. Reported values are averages of five samples with the standard deviation shown as error bars. *p < 0.05 (*t* test).

These findings show that the rheological properties of nanocellulose reinforced hydrogels can be fine-tuned by changing the concentrations of either HA-PEG or nanocellulose. In addition, the mechanical properties of the hydrogels could also be modified either by addition of Ca^2+^ to increase *G*′ or cellulase to decrease *G*′. The oxalate side groups on the cellulose can coordinate with Ca^2+^ ions, resulting in ionic cross-linking of the nanocellulose. Both the storage modulus and viscosity of the nanocellulose containing hydrogels increased by approximately 12% when immersed in CaCl_2_, whereas this change was not observed for hydrogels without nanocellulose (Figure S3). These hydrogels contained 0.5% (w/v) NC_0_, and the effect of Ca^2+^ addition would likely be even more pronounced with higher nanocellulose content. Cellulases are a group of enzymes with the ability to catalyze the hydrolysis of cellulose macromolecules.^34^ Immersion of a nanocellulose containing HA-PEG hydrogel in a 3.3 mg/mL cellulase solution resulted in a reduction of the hydrogel storage modulus with more than 50% (Figure 4). The difference between the storage modulus values before and after immersion in cellulase solution was statistically significant for the sample containing cellulose (*t*(4) = 4.72, *p* = 0.009), but not for the sample without cellulose.

**Figure 5:**
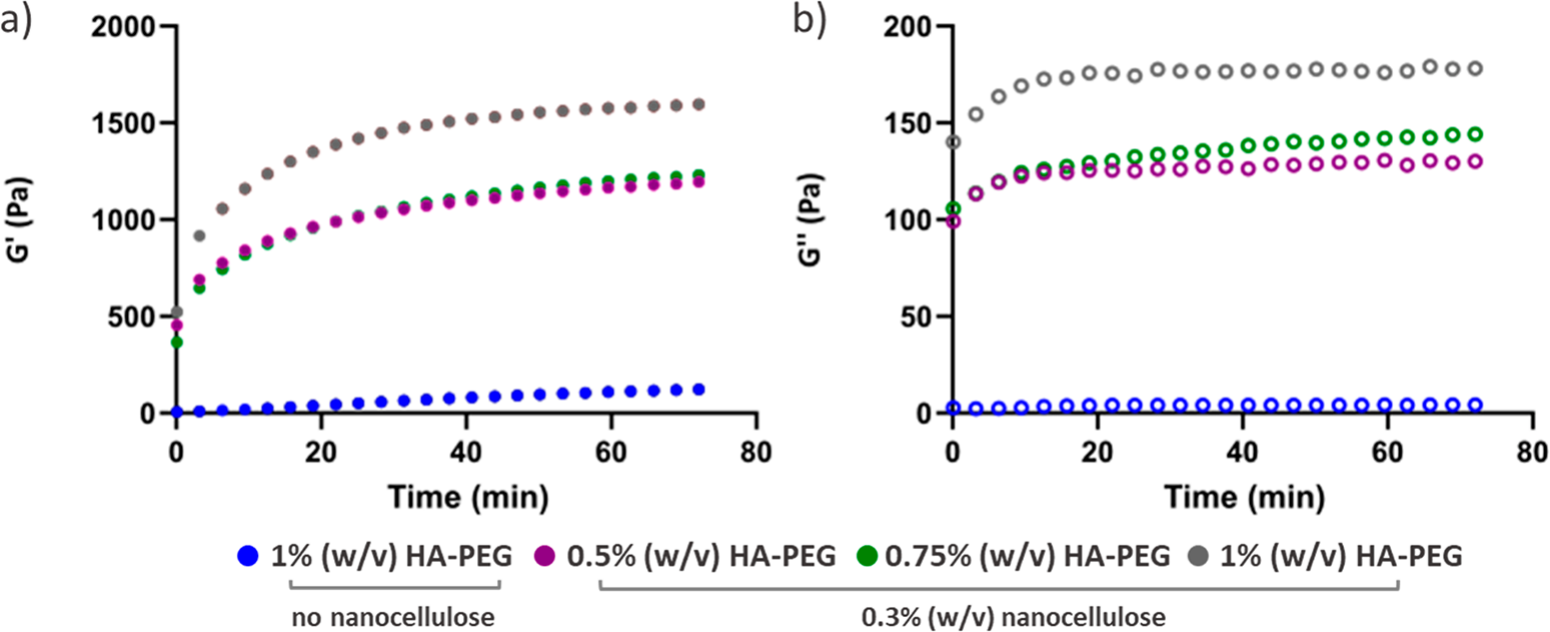
Gelation kinetics for 0.5, 0.75 and 1% (w/v) HA-PEG hydrogels containing either no cellulose or 0.3% (w/v) NC_0_: (a) storage modulus; (b) loss modulus

The addition of nanocellulose to the HA-PEG hydrogels had a pronounced effect on the gelation kinetics (Figure 5). The rate of gelation for the nanocellulose containing hydrogels was markedly higher during the first 20 min compared to hydrogels without nanocellulose. The cellulose fibrils likely become physically trapped within the HA-PEG network during the cross-linking reaction, resulting in a faster increase in storage modulus and viscosity despite the actual covalent cross-linking taking place at a similar rate in the samples with and without nanocellulose. The storage and loss moduli reached higher values during the gelation kinetics measurements than during measurements on preformed hydrogels at the same dry content of nanocellulose and HA-PEG, since the preformed hydrogels were evaluated after swelling in PBS overnight. The effect of swelling in PBS on storage and loss moduli was more pronounced for the nanocellulose containing hydrogels than for hydrogels consisting of HA-PEG alone. The viscosity of the nanocellulose relies on short-range interactions such as van der Waals forces and hydrogen bonding between the cellulose molecules.^35^ It is likely that a certain degree of swelling of the hydrogel therefore has a higher effect on the rheological properties of the cellulose component than on the covalently cross-linked HA-PEG.

**Figure 6:**
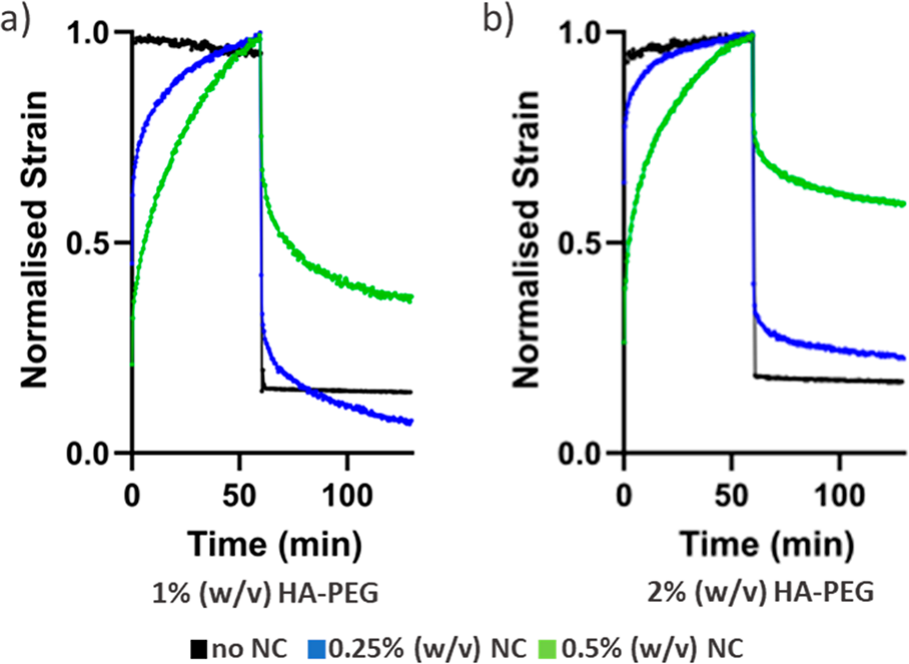
Normalized strain showing the deformation and recovery after creep-recovery test of (a) 1% (w/v) HA-PEG containing either 0.5% or 0.25% (w/v) NC_0_ and (b) 2% (w/v) HA-PEG containing either 0.5% or 0.25% (w/v) NC_0_.

Creep-recovery tests (Figure 6) showed that a higher nanocellulose content in the hydrogels increased the permanent deformation of the hydrogels after stress had been applied and released due to rearrangement of the nanocellulose fibrils under stress. A higher permanent deformation corresponds to a material with a higher plasticity, which can have a large impact on cell–biomaterial interactions.^36,37^

**Figure 7:**
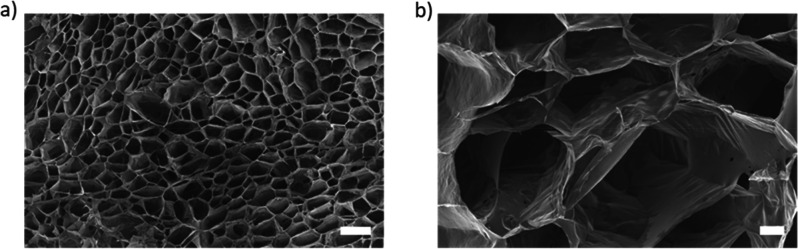
SEM of lyophilized samples of 1% (w/v) HA-PEG containing 0.5% (w/v) NC_0_: (a) 250×, scale bar: 100 μm; (b) 1000×, scale bar: 20 μm.

To investigate the microarchitecture of the hydrogels, samples composed of 1% (w/v) HA-PEG and 0.5% (w/v) NC_0_ were imaged by SEM (Figure 7). The samples were frozen by immersion in N_2_(*l*) and lyophilized prior to imaging. The hexagonally shaped pores are likely an effect of the freezing procedure. The morphology on SEM was similar for hydrogels with and without nanocellulose (Figure S4).

### Cell Viability

**Figure 8:**
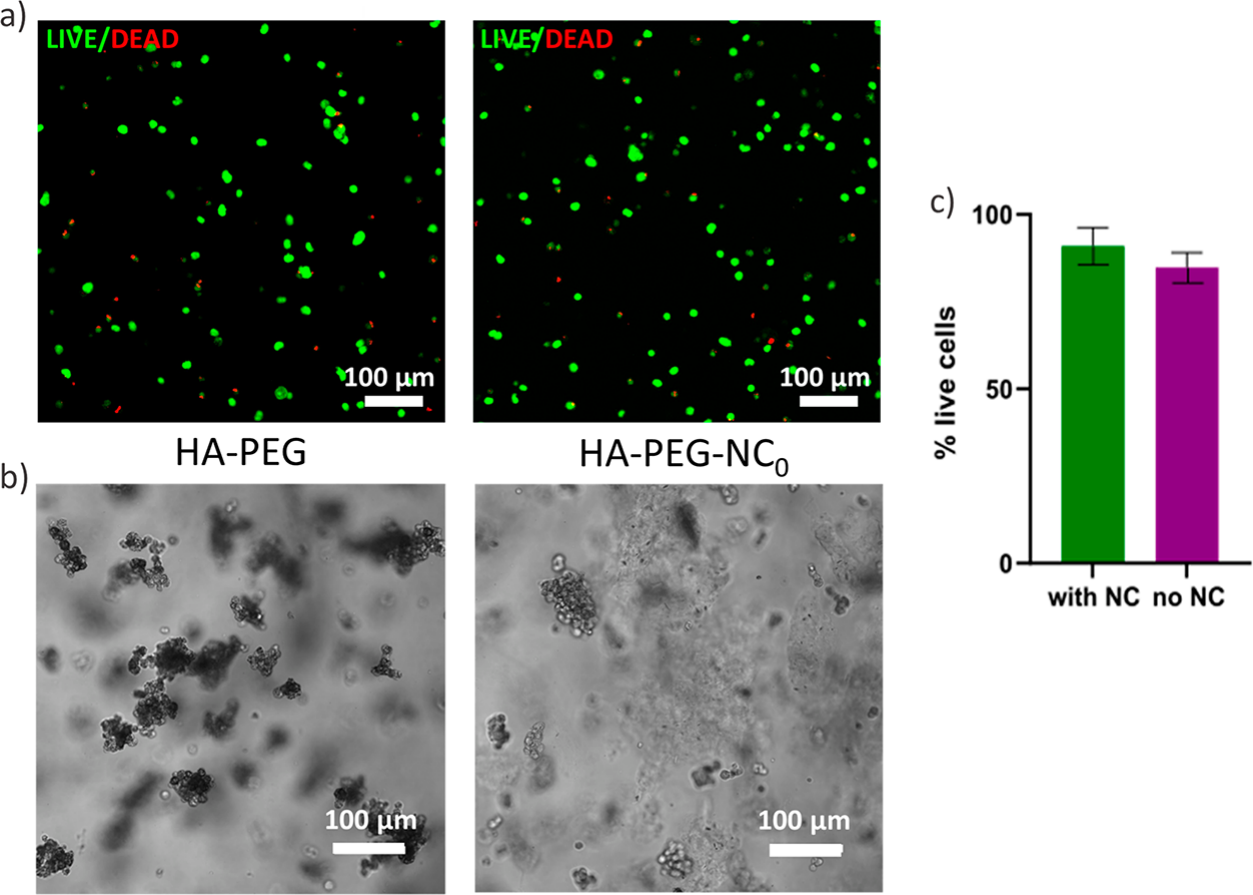
3D cultured primary human fibroblasts in a 2% (w/v) HA-PEG hydrogel with and without 0.5% NC_0_. For each sample, a minimum of five replicates was made, and each condition was evaluated in two parallel experiments. (a) Confocal microscopy images with Live/Dead staining at day 7, left: without NC_0_, right: with NC_0_. (b) Light microscopy images of 3D cultured fibroblasts at day 7 after seeding: left, without NC_0_; right, with NC_0_. (c) Quantification of live cells based on confocal images of Live/Dead stained cells day 7, standard deviation is shown as error bars.

To assess the cytocompatibility of the hydrogels, we encapsulated and cultured both a neuroblastoma cell line (SH-SY5Y) and primary human dermal fibroblasts in the hydrogels. The relatively delicate cell line SH-SY5Y was used to evaluate any possible differences in cell viability when cultured in hydrogels containing nanocelluloses of different charge densities. No significant difference in cell viability were seen for SH-SY5Y cultured in hydrogels containing either NC_0_, NC_15_, or NC_60_ (Figure S5), for which reason subsequent experiments were performed only with NC_0_. Fibroblasts were seeded in 2% (w/v) HA-PEG with and without 0.5% (w/v) NC_0_. Live/dead assay indicated high viability for both conditions at day 7 (Figure 8) with no significant difference with and without nanocellulose (*t*(6) = 1.85, *p* = 0.11). The rounded fibroblast morphology is likely a result of the lack of cell-adhesion motifs in the hydrogels. We have previously demonstrated that cell adhesions motifs, such as the fibronectin and laminin derived peptides RGD and IKVAV, with terminal azide groups can be easily conjugated to the HA-BCN backbone, resulting in improved cell–hydrogel interactions.^8^

### Printability

**Figure 9:**
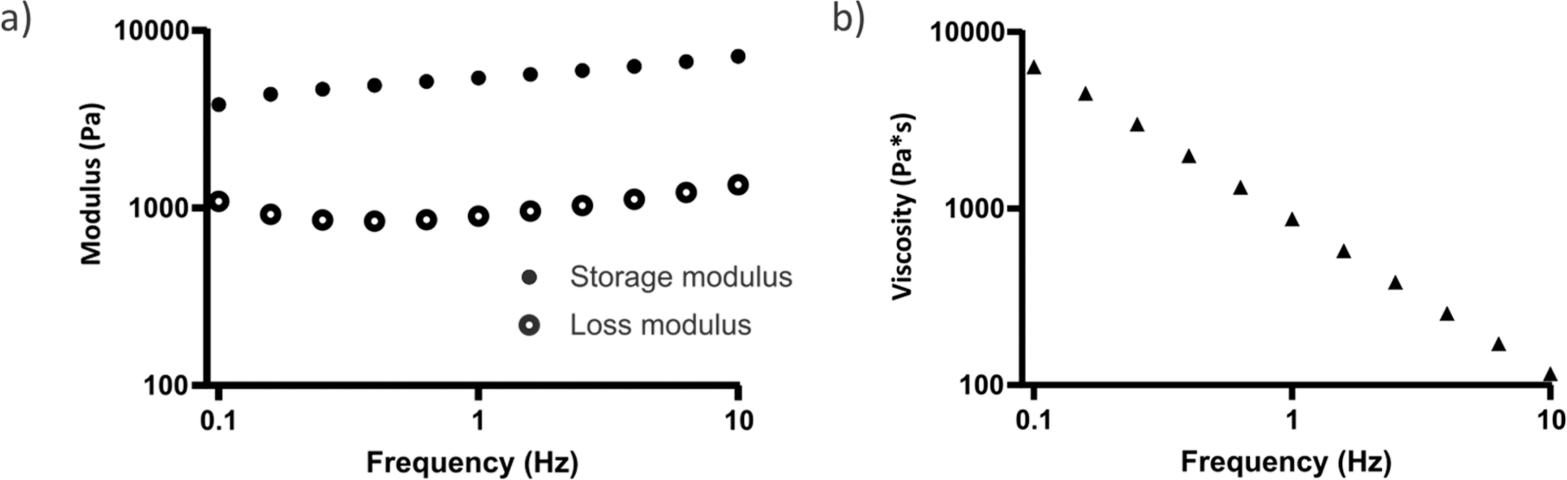
(a) Storage and loss modulus and (b) complex viscosity of 1% (w/v) HA-BCN with 1.3% (w/v) NC_0_.

**Figure 10:**
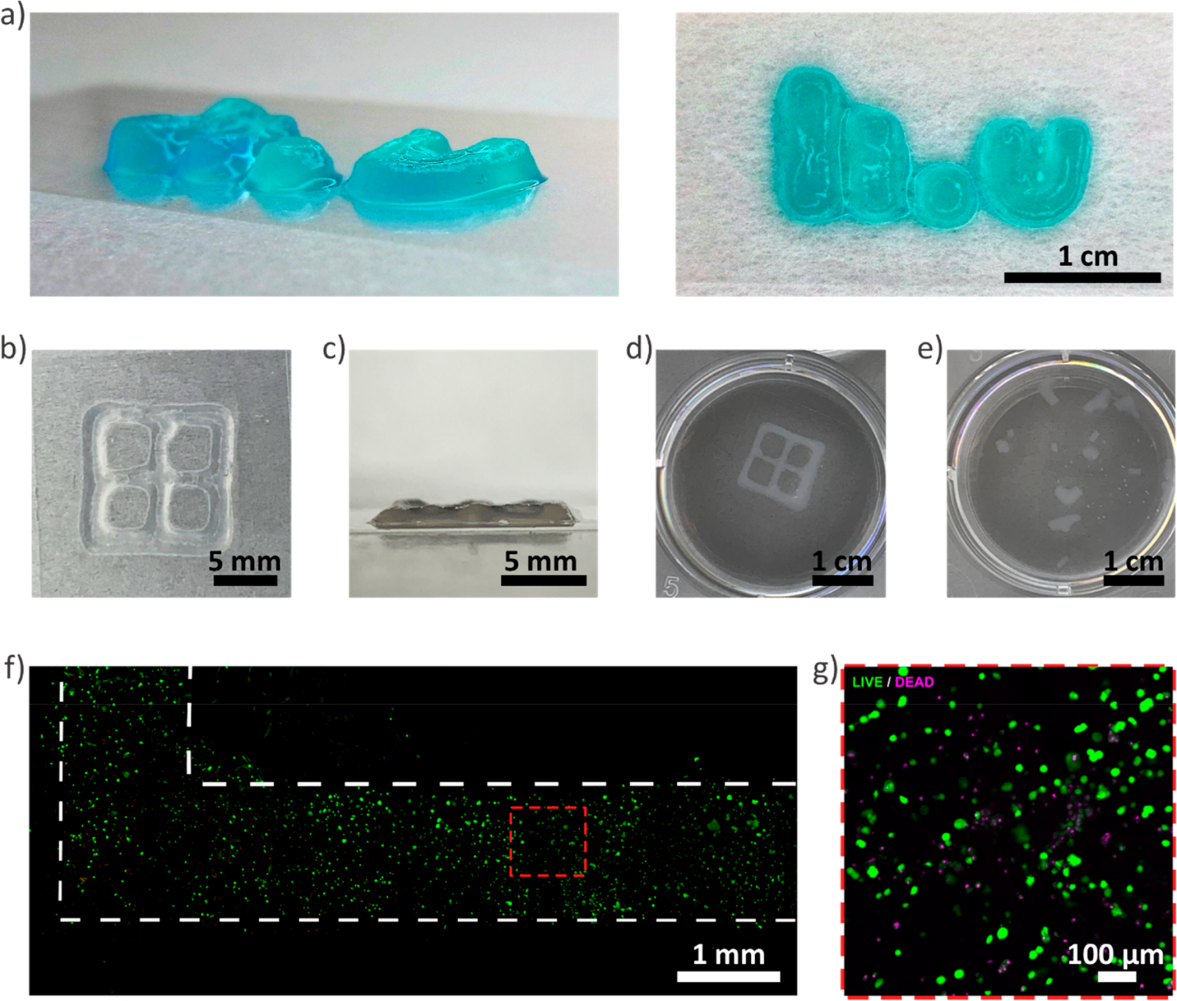
(a) A printed LiU logo (with permission from Linköping University) after cross-linking. The structure was dyed with a food colorant for increased visibility. (b) Optical microscopy images of top and (c) side view of a printed lattice structure. (d) The cross-linked structure remained intact overnight at 37 °C in PBS. (e) Printed structures without added cross-linker had disintegrated after an overnight incubation at 37 °C in PBS. (f) Confocal micrograph of a printed lattice structure with a fibroblast containing bioink with Live/Dead staining. (g) Confocal micrograph of Live (green)/Dead (red) staining shows that the viability of the cells was 83% 24 h after printing.

Shear thinning hydrogels are attractive as bioinks for extrusion-based 3D bioprinting since this facilitates the dispensing procedure while allowing for printing with high shape fidelity. Addition of nanocellulose (1.3% w/v) to HA-BCN (without PEG-Az_8_) resulted in an increase in storage modulus and viscosity of several orders of magnitude, from ∼3 to ∼5700 Pa and ∼0.5 to ∼900 Pa·s, respectively (Figure 9, Table 1). Thanks to the high storage modulus and shear thinning behavior of this bioink, printing of self-supporting structures was possible without addition of cross-linker prior to extrusion (Figure 10a–d). The cross-linker (PEG-Az_8_) was instead added after printing by applying PBS containing 1% (w/v) PEG-Az_8_.

**Table 1:** Rheological Properties of 1% (w/v) HA-BCN with and without NC_0_ (1.3%, w/v)

	ink with NC_0_	ink without NC_0_	NC_0_ only
*G*′ (Pa)	5720	3.1	4000
*G*′′ (Pa)	860	0.2	700
viscosity (Pa·s)	920	0.5	650
tan delta	0.15	0.08	0.17

After careful optimization of printing parameters, high aspect ratio self-supported multilayer structures could be printed (Figure 10a). After the addition of a cross-linker, printed structures were stable and immersed in PBS (Figure 10b,c), while printed structures that were not cross-linked disintegrated under the same conditions (Figure 10d,e). The shear thinning properties combined with the biorthogonal postprinting cross-linking further ensured that cells retained high viabilities. The viability of the primary human fibroblast printed with this bioink after 24 h was estimated to be 83% (Figure 10f,g).

## Conclusions

This study demonstrates the successful incorporation of a nanocellulose oxalate dispersion into a hyaluronan-based bioink. The incorporation of nanocellulose resulted in a hydrogel with excellent printability that could be cross-linked post extrusion using a bioorthogonal SPAAC reaction. The nanocellulose further offered the option of either cross-linking the cellulose oxalate with Ca^2+^ to increase storage modulus, or to decrease storage modulus by addition of cellulase which degraded the cellulose component of the hydrogels. The charge density of the cellulose oxalate had little to no effect on rheological properties or cell viability. The rheological properties of the hydrogels could be tuned by adjusting the concentration of either HA-PEG or nanocellulose. We anticipate that this hydrogel system can find broad use as a bioink to produce complex geometries which can support cell growth.

## Supporting Information Available

Frequency sweeps for data presented in Figure 3; Rheological properties of hydrogels before and after addition of CaCl_2_; SEM micrographs of lyophilized HA-PEG hydrogels; Viability of SH-SY5Y cultured in hydrogels containing either NC_0_, NC_15_ or NC_60_; AlamarBlue data related to Figure 8; Quantification of the swelling of hydrogels after storage in PBS overnight compared with immediately after cross-linking (PDF)
