# Supplementary material for: Nanocellulose Reinforced Hyaluronan-Based Bioinks
Source: Biomacromolecules. 2023 Jun 21;24(7):3086–93. doi: 10.1021/acs.biomac.3c00168 (PMC10336840; doi:10.1021/acs.biomac.3c00168)
Supplement: Supplementary file 1 — bm3c00168_si_001.pdf [file bm3c00168_si_001.pdf]

Supporting information

Nanocellulose Reinforced Hyaluronan Based Bioinks

*Andrea Träger, Sajjad Naemipour, Michael Jury, Robert Selegård, Daniel Aili**

Laboratory of Molecular Materials, Division of Biophysics and Bioengineering, Department of Physics, Chemistry and Biology, Linköping University, 581 83 Linköping, Sweden.

*Corresponding author, email: daniel.aili@liu.se

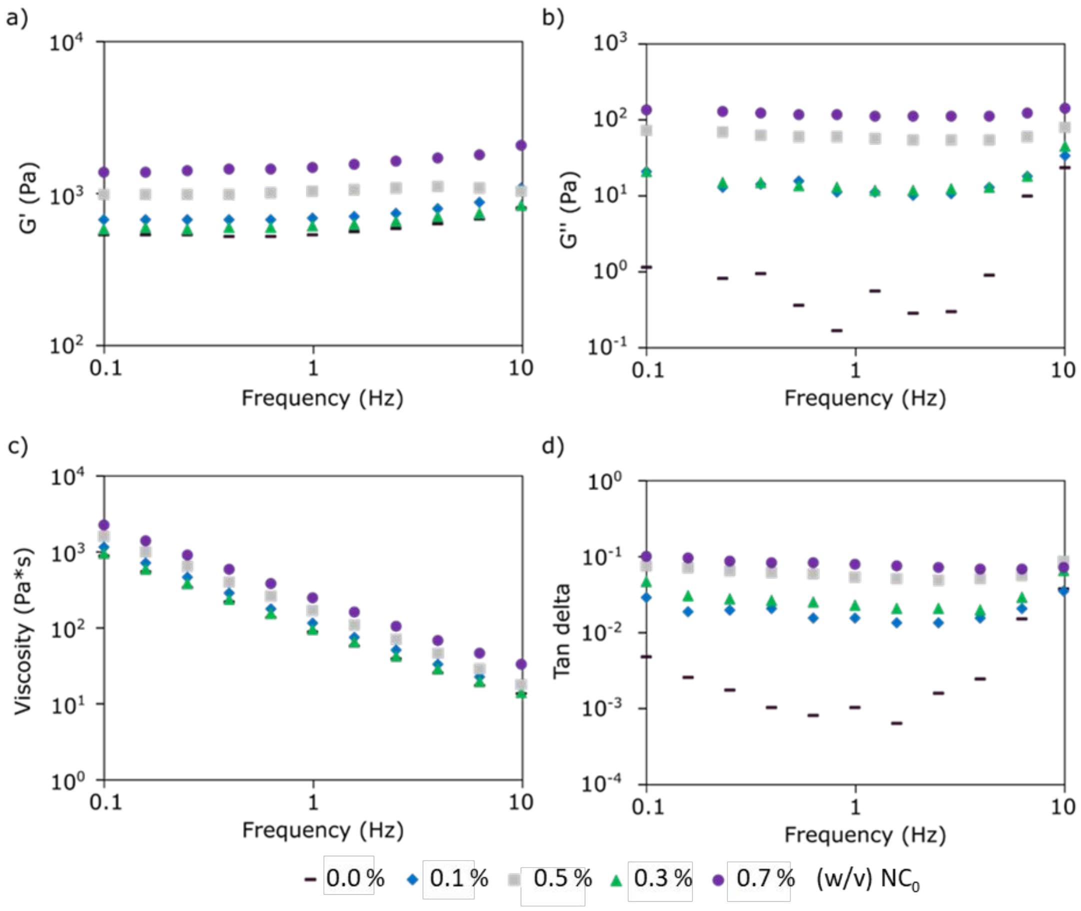

Figure S1. Rheological properties of 2% (w/v) HA-PEG hydrogels containing between 0 and 0.7% (w/v) of NC_0. a) storage modulus b) loss modulus c) complex viscosity d) tan delta. Reported values are averages of at least four measurements, standard deviation shown as error bars.

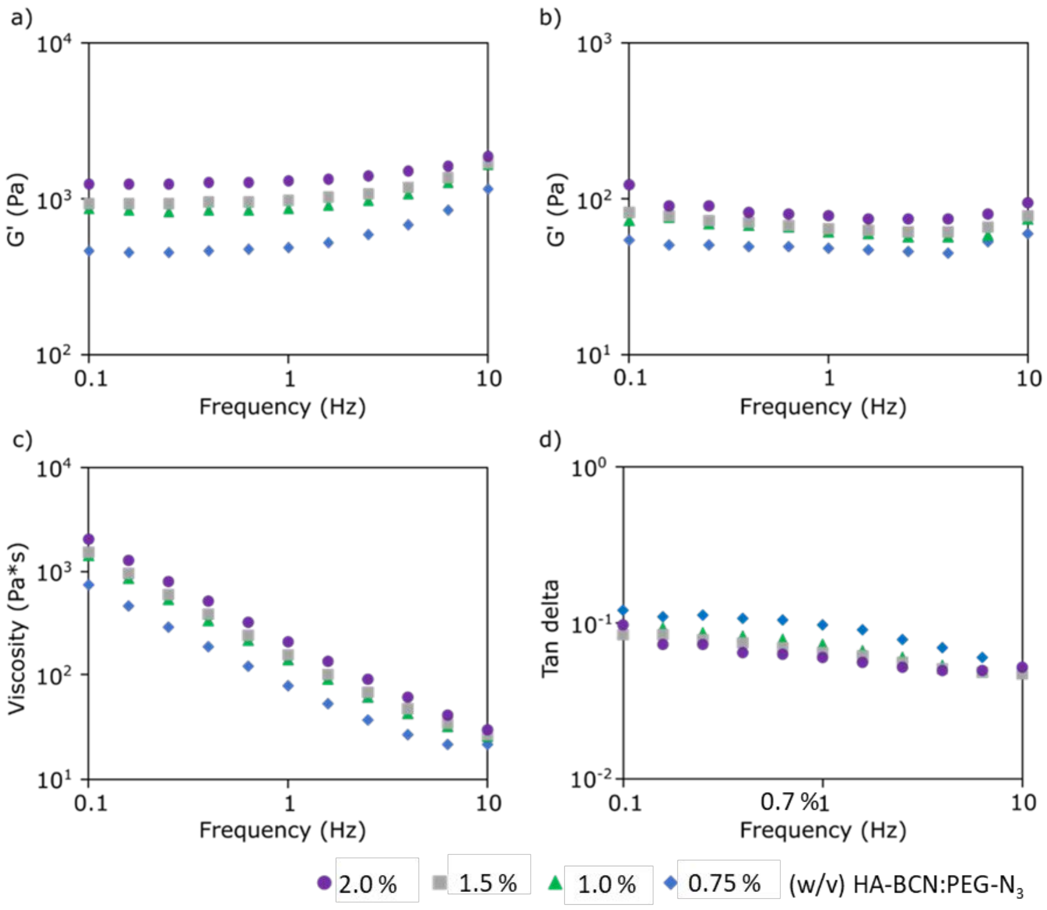

Figure S2. Rheological properties of hydrogels containing 0.5% (w/v) NC_0 and different concentrations of HA-PEG. a) storage modulus b) loss modulus c) complex viscosity d) tan delta. Reported values are averages of at least four measurements, standard deviation shown as error bars.

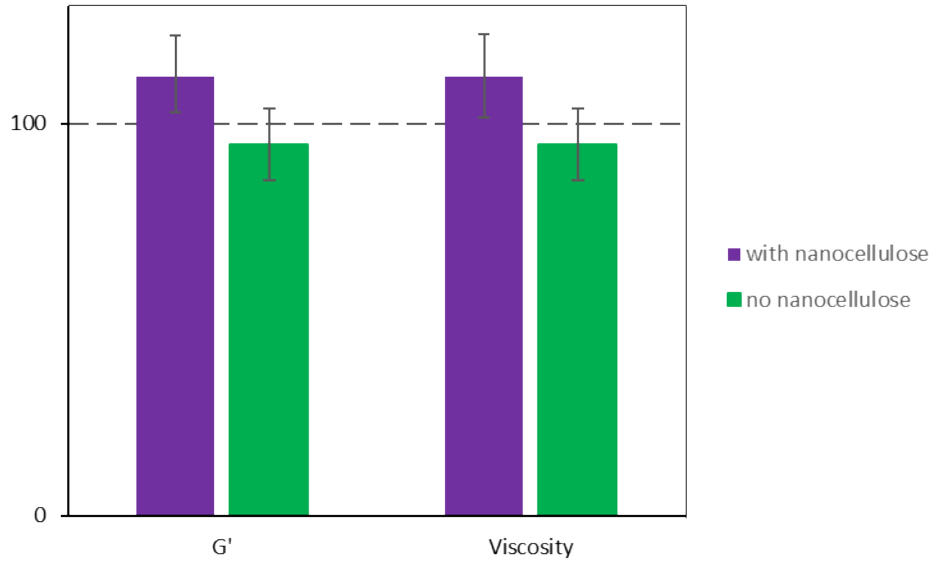

Figure S3. Relative change in percent of storage modulus and viscosity of 0.75% (w/v) HA:PEG hydrogels containing 0.5% (w/v) NC_0, after immersion in 77 mM $CaCl_2$ relative to previous immersion in NaCl of the same ionic strength. Reported values are averages of at least 6 replicates with standard deviation shown as error bars. Each individual sample was first immersed in the NaCl solution for 24h before rheological measurement, then transferred to the $CaCl_2$ solution for 24h before a second rheological measurement. The difference in storage modulus and viscosity at 1Hz frequency and 1% oscillation strain after immersion in each solution was calculated for each individual sample, and then averaged.

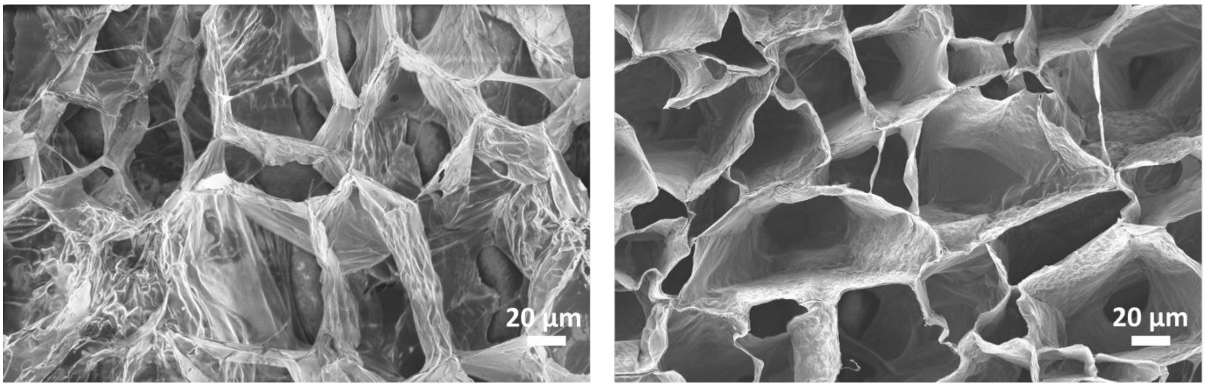

Figure S4. SEM images of lyophilised HA-PEG hydrogels. Left: 1 % (w/v) HA-PEG Right: 2 % (w/v) HA-PEG.

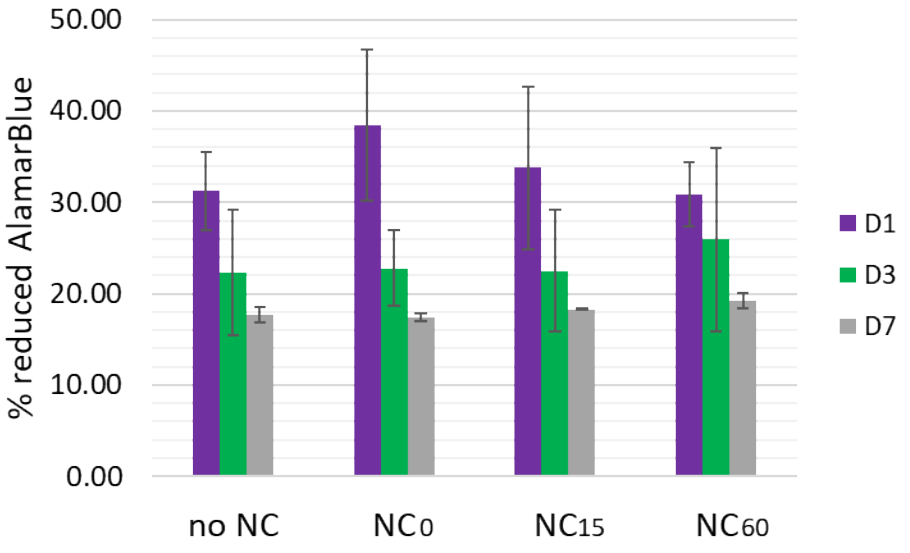

Figure S5. Percentage reduced AlamarBlue by SH-SY5Y cells days 1, 3 and 7 after seeding in 1 w/w% HA-PEG containing either no cellulose or 0.5 w/w% of NC_0, NC_{15} or NC_{60}, standard deviation shown as error bars.

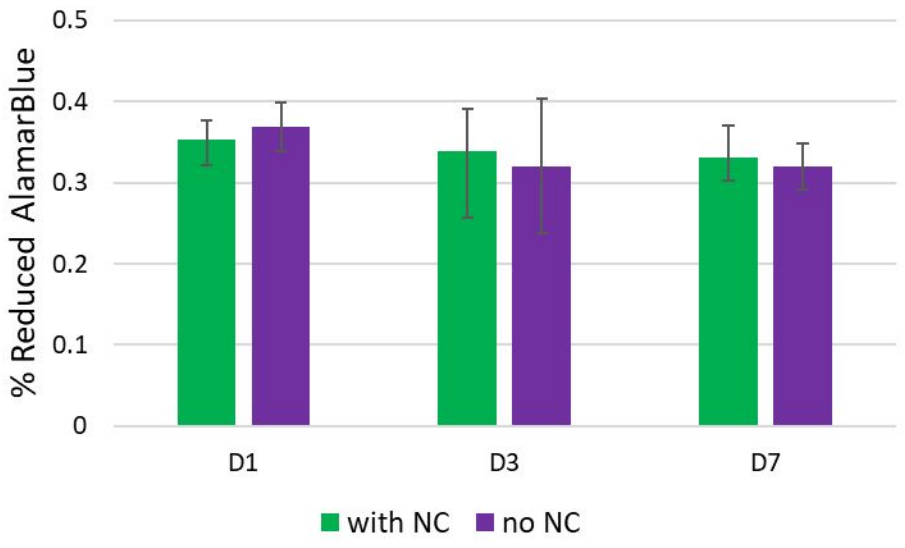

Figure S6. Percentage reduced AlamarBlue by human primary fibroblasts days 1, 3 and 7 after seeding in 2 w/w% HA-PEG with and without 0.5 % NC_0, standard deviation shown as error bars.

Table S1. Percentage of original weight of 1% (w/v) HA-PEG hydrogels containing 0-0.5% (w/v) NC_0 after storage in PBS overnight, compared to immediately after crosslinking.

Sample	% of original weight after storage in PBS overnight, compared with immediately after crosslinking
1% HA-PEG with 0.5% NC_0	112
1% HA-PEG with 0.25% NC_0	141
1% HA-PEG with no NC	155

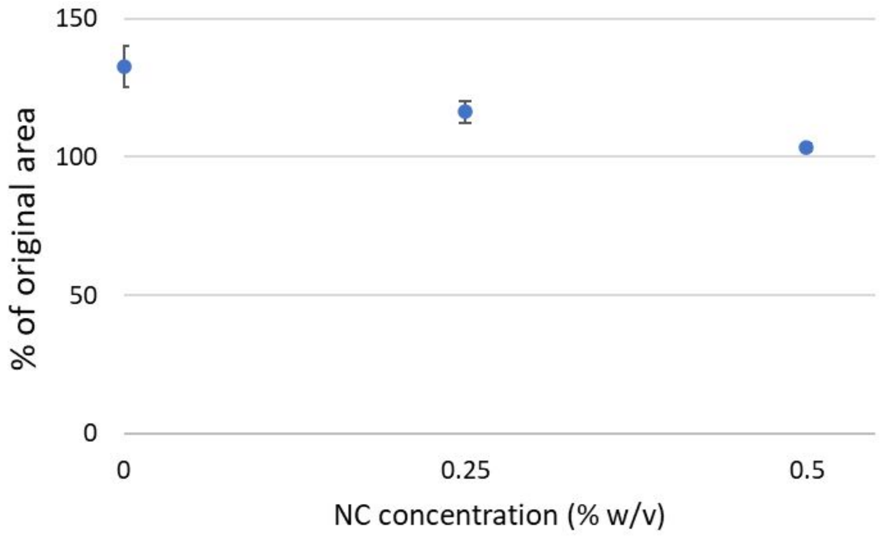

Figure S7. Percentage of original area after storage in PBS overnight compared with immediately after crosslinking of 1% HA-PEG hydrogels containing 0-0.5% (w/v) NC_0. The hydrogels were photographed together with a ruler immediately after crosslinking and again after storage in PBS overnight, and the area of each hydrogel was quantified using ImageJ. Reported values are averages of three replicates with standard deviation shown as error bars.
